# Room-temperature strong coupling in a single-photon emitter-metasurface system

**DOI:** 10.1038/s41467-024-46544-w

**Published:** 2024-03-13

**Authors:** T. Thu Ha Do, Milad Nonahal, Chi Li, Vytautas Valuckas, Hark Hoe Tan, Arseniy I. Kuznetsov, Hai Son Nguyen, Igor Aharonovich, Son Tung Ha

**Affiliations:** 1 Institute of Materials Research and Engineering, A*STAR (Agency for Science, Technology and Research), Singapore, 138634 Republic of Singapore; 2 School of Mathematical and Physical Sciences, Faculty of Science, University of Technology Sydney, Ultimo, NSW 2007 Australia; 3 ARC Centre of Excellence for Transformative Meta-Optical Systems, Faculty of Science, University of Technology Sydney, Ultimo, NSW 2007 Australia; 4 Department of Electronic Materials Engineering, Research School of Physics and Engineering, The Australian National University, Canberra, ACT 2600 Australia; 5 ARC Centre of Excellence for Transformative Meta-Optical Systems, Research School of Physics and Engineering, The Australian National University, Canberra, ACT 2600 Australia; 6 Univ Lyon, Ecole Centrale de Lyon, CNRS, INSA Lyon, Universite Claude Bernard Lyon 1, CPE Lyon, CNRS, INL, UMR5270, 69130 Ecully, France; 7 Institut Universitaire de France (IUF), F-75231 Paris, France; 8 Present Address: Department of Chemistry, The University of Manchester, Oxford Road, Manchester, M13 9PL UK; 9 Present Address: School of Physics and Astronomy, Monash University, Melbourne, VIC 3800 Australia

**Keywords:** Quantum optics, Single photons and quantum effects

## Abstract

Solid state single-photon sources with high brightness and long coherence time are promising qubit candidates for modern quantum technology. To prevent decoherence processes and preserve the integrity of the qubits, decoupling the emitters from their surrounding environment is essential. To this end, interfacing single photon emitters (SPEs) with high-finesse cavities is required, especially in the strong coupling regime, when the interaction between emitters can be mediated by cavity fields. However, achieving strong coupling at elevated temperatures is challenging due to competing incoherent processes. Here, we address this long-standing problem by using a quantum system, which comprises a class of SPEs in hexagonal boron nitride and a dielectric cavity based on bound states in the continuum (BIC). We experimentally demonstrate, at room temperature, strong coupling of the system with a large Rabi splitting of ~4 meV thanks to the combination of the narrow linewidth and large oscillator strength of the emitters and the efficient photon trapping of the BIC cavity. Our findings unveil opportunities to advance the fundamental understanding of quantum dynamical system in strong coupling regime and to realise scalable quantum devices capable of operating at room temperature.

## Abstract (web summary)

Interfacing single-photon emitters (SPEs) with high-finesse cavities can prevent decoherence processes, especially at elevated temperature, but its implementation remains challenging. Here, the authors report room-temperature strong coupling of SPEs in hexagonal boron nitride with a dielectric cavity based on bound states in the continuum, showing a Rabi splitting of ~ 4 meV.

## Introduction

Cavity quantum electrodynamics (cQEDs) describes coupled systems comprising of optically active emitters with atom-like transitions, such as single-photon emitters (SPEs) and optical cavities. Single-photon emitters (SPEs) can act as elementary blocks (qubits) for solid-state quantum computers, and their interaction can be mediated by cavity electric fields^1^. In most cases, SPE-cavity systems undergo incoherent processes of weak coupling, in which the fluorescence is enhanced by the Purcell effect. As a result, weak coupling can expedite spontaneous emission, thereby enhancing the indistinguishability and augment the collection efficiency of the emission through outcoupling engineering. On the other hand, in the strong coupling regime, emitters and photons exchange energy coherently, leading to two new hybrid half-light-half-matter states (polaritons) separated by a characteristic Rabi splitting energy $2g$, where $g$ is the coupling strength. The formation of two new energy states in two-level SPEs, or Jayne-Cumming ladders in multi-level SPEs, demonstrate prominent anharmonicity, which can be harnessed to establish a photon-blockade regime, thereby allowing for the on-demand regulation of the number of photons transmitted under resonant excitation^2–4^. Furthermore, cQEDs in strong coupling regime also provides a promising platform for distributing coherent interaction and entangled links among the quantum nodes via external control fields. Several cQED-based schemes have been proposed for quantum information processing^5–8^.

The relationship between the coupling strength $g$ and the optical transition dipole moment ${{{{{\boldsymbol{\mu }}}}}}$, local electric field ${{{{{\bf{E}}}}}}$, oscillator strength $f$ and cavity mode volume $V$ can be expressed as:

1 $$g={{{{{\boldsymbol{\mu }}}}}}.{{{{{\bf{E}}}}}}={\hslash }\sqrt{\frac{\pi {e}^{2}f}{4{\epsilon }_{r}{\epsilon }_{0}{m}_{0}V}},$$

where ${\epsilon }_{r}{\epsilon }_{0}$ is the dielectric constant of the cavity material and ${m}_{0}$ is the free electron mass. Theoretically, the condition $g\ge |{\kappa }_{{{{{{\rm{cav}}}}}}}-{\kappa }_{{{{{{\rm{SPE}}}}}}}|/2$, where ${\kappa }_{{{{{{\rm{cav}}}}}}}$ and ${\kappa }_{{{{{{\rm{SPE}}}}}}}$ are dissipative decay rates of the cavity and the emitter, respectively, guarantees real solutions for two hybrid eigenstates^9^. This requirement can be fulfilled by (i) enhancing $g$ via reducing $V$, increasing $f$ and/or (ii) lowering the critical threshold for coupling strength via using narrow-line emitters (small ${\kappa }_{{{{{{\rm{SPE}}}}}}}$) and high-finesses cavities (high quality ($Q$) factor, small ${\kappa }_{{{{{{\rm{cav}}}}}}}$). Unlike excitons in three-dimensional and two-dimensional systems, transition dipole moments of single emitters are generally small leading to small coupling strength $g$ in SPE-cavity systems. Therefore, going down to the level of single-photon emission for strong coupling has been a long-standing challenge, especially at elevated temperatures when the incoherent processes become more pronounced and the radiation loss ${\kappa }_{{{{{{\rm{SPE}}}}}}}$ is substantially enhanced due to the interaction with the phonon bath. Strong coupling emission of solid-state SPEs has been experimentally observed in high-$Q$ cavities at cryogenic temperature^10–14^. Exploring new lossless systems consisting of narrow-line SPEs and lossless cavities is a viable route to push such quantum system towards strong coupling at room temperature.

Substantial efforts have been devoted to fabricating optical cavities sustaining both high-$Q$ and small-$V$ simultaneously^15–18^. The $Q$-factor of these cavities is very sensitive to the fabrication imperfection. Furthermore, the small mode volume imposes a serious challenge for precisely positioning a single emitter at the electric-field maxima within such small active mode volumes and aligning emitter dipole moments with cavity fields. Therefore, the design of optical cavities and the choice of SPE sources become critical to satisfy the strong coupling criteria.

Here, we demonstrate room-temperature strong coupling of SPEs in a high-*Q*, large-$V$ cavity using the concept of bound-state in-the continuum (BIC). In theory, photons are perfectly trapped inside BIC cavities^19^ (${\tau }_{{{{{{\rm{BIC}}}}}}}\to \infty$) and the condition reduces to $g\ge {\kappa }_{{{{{{\rm{SPE}}}}}}}/2$, since ${\kappa }_{{{{{{\rm{BIC}}}}}}}={\tau }_{{{{{{\rm{BIC}}}}}}}^{-1}$ equals 0. Therefore, the strong coupling is no longer restricted by the cavity loss. Such an ultrahigh-*Q*-factor of the symmetry-protected BIC is very robust against minor fabrication imperfections as long as the overall symmetry is preserved that is a big advantage of using BIC cavity over other types such as dielectric photonic crystals or plasmonic cavities. BIC plays a vital role in the recent progress in nonlinear optics^20–22^, functional metasurface^23^, lasing^24–27^, and Bose-Einstein condensation^28^. SPEs used in this work are carbon-based colour centres generated in a few-layer-thick hexagonal boron nitride (hBN) film, which are reported to have bright emission, non-blinking nature, ultrahigh Debye-Wallor factor (${F}_{{{{{{\rm{DW}}}}}}}$~0.82), optically addressable spin states, and high single-photon purity at high temperatures up to 800 K^29,30^. Most importantly, their emission has uniquely narrow linewidth at room temperature, which can even reach the Fourier-transform limit (i.e., ${\kappa }_{{{{{{\rm{SPE}}}}}}}$~0.2 meV)^31^, greatly favouring for strong coupling. In our SPE-BIC systems, we resolved a Rabi splitting of ~4 meV at room temperature and at single-photon emitter levels. Our results strongly suggest that the combination of BIC cavities and SPEs in two-dimensional materials is a viable route towards scalable quantum devices operating at ambient conditions.

## Results

### Device concept and fabrication

**Fig. 1:**
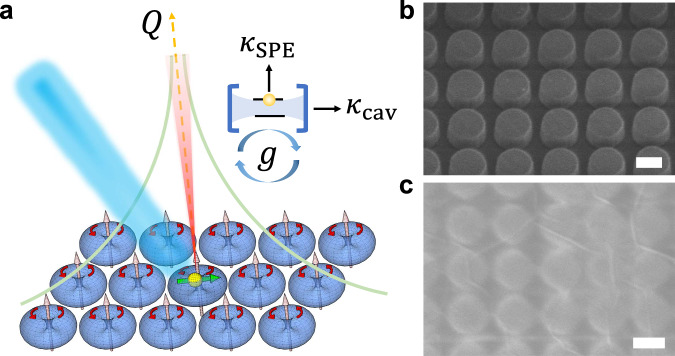
Single-photon emitter in the bound-state-in-the-continuum cavity. **a** Illustration of coupling between a single-photon emitter (SPE) and a bound-state-in-the-continuum (BIC) cavity with quality factor $Q$. The BIC mode is formed by an array of vertical magnetic dipoles (pink arrows), which associates with an electric-field (blue doughnuts) circulating in the *x*-*y* plane (red arrows). An SPE with a horizontal transition dipole moment (yellow sphere with green arrow) spatially overlaps with the electric-field hotspots. For strong coupling, the theoretical condition $g\ge |{\kappa }_{{{{{{\rm{cav}}}}}}}-{\kappa }_{{{{{{\rm{SPE}}}}}}}|/2$ has to be satisfied, where $g$ is the coupling strength, ${\kappa }_{{{{{{\rm{cav}}}}}}}$ and ${\kappa }_{{{{{{\rm{SPE}}}}}}}$ are dissipative decay rates of the cavity and the emitter, respectively. **b** 30° tilted scanning electron microscope (SEM) image of the fabricated TiO_2_ nanopillar array (diameter $\sim$ 260 nm, gap $\sim$4 nm). **c** SEM image of the array after transferring a 3-nm thick hexagonal boron nitride (hBN) film on top. Scale bars in (**b**) and (**c**) are 200 nm.

Figure 1a illustrates the concept of coupling between a single-photon emitter and a BIC cavity formed by a sub-diffractive square array of dielectric resonators. Due to the system symmetry, the total destructive interference of the vertical magnetic dipoles from all resonators (pink arrows) leads to the formation of BIC at normal incident angle. The SPE (yellow sphere) is located within the electric field $({{{{{\bf{E}}}}}})$ distribution (blue doughnuts), and the transition dipole moment (${{{{{\boldsymbol{\mu }}}}}}$) of the emitter (green arrow) is randomly aligned. The coupling strength $g\propto {{{{{\boldsymbol{\mu }}}}}}{{{{{\boldsymbol{.}}}}}}{{{{{\bf{E}}}}}}$ is optimal when the dipole is completely aligned with the local field **E**.

In particular, our BIC cavity is constructed by a 50 × 50 µm^2^ square array of nanopillars made of titanium dioxide (TiO_2_) as shown in the scanning electron microscope (SEM) image in Fig. 1b. Being a relatively high refractive index (i.e., $n$~2.5 at ~600 nm) and lossless material at optical frequencies, TiO_2_ nanostructures manifest themself as ideal candidates for an ultrahigh-*Q* cavity that have been demonstrated to support BIC modes originated not only from vertical magnetic dipole but also from vertical electric dipoles, and electric quadrupoles^26^. Here, the nanopillar diameter $(D)$ and their gap are varied to tune the resonance position of the vertical magnetic dipole BIC mode to spectrally overlap with the emission from SPEs. A 3-nm-thick hBN film (Supplementary Fig. 1) was grown by metal-organic vapour-phase epitaxy on a sapphire substrate and then transferred onto the fabricated TiO_2_ nanostructures (Fig. 1c). The SPEs are subsequently activated by a thermal annealing process (see “Methods” section).

### Characteristics of SPEs and BIC cavity

**Fig. 2:**
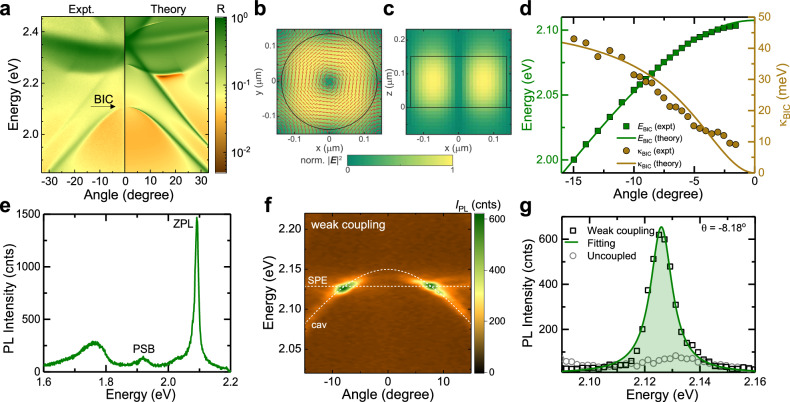
Optical characterisation for the BIC cavity, SPE and their weak coupling. **a** Experimental (left) and calculated (right) angle-resolved unpolarised reflectance spectra of the designed TiO_2_ nanopillar array showing vertical magnetic dipole BIC resonance at ${E}_{{{{{{\rm{BIC}}}}}}} \sim$ 2.107 eV at angle $\theta=$ 0°. **b** Top view and **c** side view of the electric-field distribution at the BIC frequency in the nanopillars. The direction of the electric field is represented by red arrows. **d** Experimental resonance energies (green squares) and full-width at half-maximum (FWHM) (light-brown circles) of the BIC mode observed in (**a**) as a function of $\theta$. The corresponding fitting curves (solid lines) are obtained using the BIC model discussed in the SI. **e** Typical photoluminescence (PL) spectrum of carbon-related SPEs showing the zero-phonon-line (ZPL) peak at $\sim$2.092 eV, and a phonon side-band (PSB) at $\sim$1.919 eV. **f** Angle-resolved PL spectra in a weak coupling regime, showing a PL enhancement of the SPE. The cavity mode and the SPE PL position are shown by the white dashed lines. **g** PL spectra of the coupled SPE extracted from (**f**) at −8.18° (black squares) and the uncoupled SPE (grey circle) under the same measurement conditions. The PL spectrum of the weakly coupled SPE is fitted by single Lorentzian (green solid line), and the PL intensity is represented by the area under the fitting curve (green shaded area).

Figure 2a shows the measured (left) and simulated (right) angle-resolved reflection spectra of the TiO_2_ nanopillar array with $D$~260 nm and gap~40 nm. As going closer to the normal incidence, the linewidth of the optical mode becomes narrower, and the intensity becomes weaker. Within the resolution limit of our setup, the smallest resolvable angle is $\sim \pm 1.5^\circ$. The spectral narrowing and the vanishing of reflectance signal when approaching normal incidence ($\theta$ = 0°) indicate the formation of the symmetry-protected BIC at ${E}_{{{{{{\rm{BIC}}}}}}}$~2.107 eV. The multipolar analysis reveals that this BIC mode originates from the vertical magnetic dipole, as reported elsewhere^26^.

The electric-field associated with the vertical magnetic dipole, simulated by rigorous coupled wave analysis (RCWA) and using the open-source S^4^ package^32^, is concentrated within the nanopillars’ peripheries in the *x*-*y* plane with the maximal field intensity at ~75 nm from the centre (Fig. 2b). Such circulating **E**-field favours for the coupling with in-plane dipoles since $g\propto {{{{{\boldsymbol{\mu }}}}}}{{{{{\boldsymbol{.}}}}}}{{{{{\bf{E}}}}}}$ (Eq. (1)). For carbon-related SPEs in a thin hBN film (~3 nm), the dipole orientation is found to be approximately in-plane with a large component ${{{{{{\boldsymbol{\mu }}}}}}}_{{{{{{\boldsymbol{/}}}}}}{{{{{\boldsymbol{/}}}}}}}$ (Supplementary Note 1). Furthermore, the field extends ~40 nm outside of the nanopillar surface along the *z*-direction (Fig. 2c), ensuring spatial overlap with SPEs in the 3-nm thick hBN film. In contrast to other high-$Q$ dielectric cavities, at BIC conditions, electric-field hotspots exist on every nanopillar, increasing the possibility of spatial overlap with randomly distributed SPEs. The total area where the electric-field strength is larger than half of the maximum value is ~68% of the whole array area (Supplementary Note 2).

The experimental angle-dependent energy $E(\theta )$ and full-width at half-maximum (FWHM) ${\kappa }_{{{{{{\rm{BIC}}}}}}}(\theta )$ of the BIC band extracted from measured reflection are shown in Fig. 2d. The fitting values (solid lines) are obtained using the BIC model (Supplementary Note 3) and assuming that the cavity is lossless (i.e., ${\kappa }_{{{{{{\rm{BIC}}}}}}}$ (0°) = 0 meV). Since the optical signal is absence at normal incidence, $Q$-factor of BIC cannot be reliably extracted from experiments. As moving away from the normal incidence, the $Q$-factor drops significantly with $Q \sim 1/{k}^{2}$, where $k$ is the photon wavevector. For a finite size two-dimensional (2D) metasurface, the momentum space is quantized as^33^

2 $${{{{{{\bf{k}}}}}}}_{{n}_{x},{n}_{y}}=\frac{{n}_{x}\pi }{{L}_{x}}\hat{{{{{{\bf{x}}}}}}}+\frac{{n}_{y}\pi }{{L}_{y}}\hat{{{{{{\bf{y}}}}}}},$$

where ${n}_{x},{n}_{y}$ are positive integers, and ${L}_{x},{L}_{y}$ are the array lengths along ${{{{{\bf{x}}}}}}$ and ${{{{{\bf{y}}}}}}$ direction, respectively. The total wavevector is formulated as $|{{{{{\bf{k}}}}}}|={({k}_{{n}_{x}}^{2}+{k}_{{n}_{y}}^{2})}^{1/2}$. Consequently, the BIC becomes a quasi-BIC, with *Q*-factor at the smallest wavevector given by ${Q}_{{BIC}}=Q({k}_{{{{{\mathrm{1,1}}}}}})$. Therefore, we estimate ${Q}_{{{{{{\rm{BIC}}}}}}} \sim 1.3\times {10}^{5}$ for our $50\times 50$ μm^2^ array (Supplementary Note 4).

Figure 2e shows a typical SPE photoluminescence (PL) spectrum at room temperature with a zero-phonon line (ZPL) at ${E}_{{{{{{\rm{SPE}}}}}}}$~2.092 eV. Upon scanning over an area of 50 × 50 mm^2^ on the as-grown hBN film, we identified 36 individual single-photon emitters (i.e., second-order correlation function at zero-time delay ${g}^{(2)}(0)$ < 0.5) with the zero-phonon line (ZPL) position ranging from ~2.026 eV to ~2.149 eV (Supplementary Fig. 2). The averagely low SPE density (~0.01 μm^−2^) increases the chance of isolating a single SPE from the surrounding SPEs with similar energies for the purer detection. In other words, it is unlikely that two similar SPEs are located within the detection spot (<38 μm^2^) of the objectives. The lifetime of these carbon-related SPEs ranges from 1.5 to 5.5 ns, while a majority of them have a lifetime of less than 3 ns (Supplementary Note 5), which is significantly shorter than other SPE sources such as nitrogen-vacancy in diamond^34^. The shorter lifetime of the SPEs indicates the higher brightness and a stronger oscillator strength that is more favourable for strong coupling.

We study the coupling of SPE and BIC modes by performing angle-resolved and energy-resolved PL measurements under non-resonance excitation (${E}_{{{{{{\rm{Exc}}}}}}}$ ~2.541 eV). Note that angle-resolved and energy-resolved spectroscopy (*e.g*., photoluminescence, reflection or transmission) carrying the information of dispersions of all photonic/excitonic bands and their interactions, is required for the study of strong light-matter interaction. Whereas only the spectral splitting observed in energy-resolved spectroscopy does not provide enough evidence for strong coupling. For example, the dip appearing in scattering spectra could be a Fano resonance resulted from the interference of a resonance with a broadband background excitation^35–38^.

Figure 2f shows the angle-resolved PL spectra of a SPE emitting at ~2.129 eV, weakly coupled with the BIC mode (${E}_{{{{{{\rm{BIC}}}}}}}$~2.149 eV) in a nanopillar array with $D$~275 nm and gap ~50 nm. Noticeably, no spectral splitting has been observed, but rather a significant enhancement of PL intensity from the coupled SPE compared to the uncoupled one (Fig. 2g). Furthermore, the BIC band dispersion remains unchanged compared to the uncoupled cavity mode (Supplementary Fig. 3a). When there is a large mismatch between ${E}_{{{{{{\rm{SPE}}}}}}}$ and ${E}_{{{{{{\rm{BIC}}}}}}}$, only weak coupling (i.e., no Rabi splitting) can be observed (Supplementary Fig. 4).

### Observation of strong coupling between SPE and BIC

**Fig. 3:**
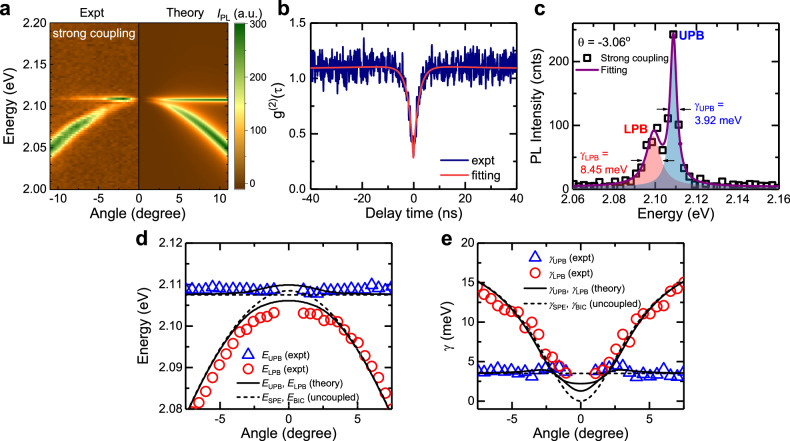
Room-temperature strong coupling in SPE-BIC systems. **a** Experimental angle-resolved PL spectra (with excitation power ${P}_{{{{{{\rm{exc}}}}}}}$~1.3^2^9 kW/cm^2^) (left) and the simulation (right) in a strong coupling regime, evidenced by the clear spectral splitting and vanishing emission at normal incidence on both polariton branches. **b** Antibunchin*g* dip *g*^2^(0) = 0.28 confirming the single-photon emission nature at the measured strong coupling spot. The experimental data (blue line) is fitted with a three-level model (red line). **c** PL spectra extracted at $\theta=$ −3.06° (black squares) and the double-Lorentzian fitting (purple curve). The FWHM of the lower polariton branch (LBP) ${\gamma }_{{LBP}}=8.45$ meV and of the upper polariton branch (UBP) ${\gamma }_{{UBP}}=3.92$ meV are extracted from the fitting. **d** Energy ($E$) and **e** FWHM $\gamma$ of the UBP (blue up-triangles) and the LBP (red circles) at different angles and the theoretical fitting using coupled oscillator model (solid black lines). The energies and FWHM of the uncoupled SPE and BIC modes are shown by dashed black lines.

When the ${E}_{{{{{{\rm{SPE}}}}}}}$ and ${E}_{{{{{{\rm{BIC}}}}}}}$ match (i.e., TiO_2_ array with $D$~260 nm and gap ~40 nm), a clear spectral splitting of the upper and lower branches can be seen, as shown in Fig. 3a. Importantly, both branches show no coupling to the radiation field at the normal incidence that inherits from the symmetry-protected BIC nature. The single-photon emission purity of the investigated emitter at the measured strong coupling spot is confirmed by the antibunching dip ${g}^{(2)}$(0) = 0.28 (Fig. 3b). By fitting the ${g}^{(2)}$ curve (red curve) with a three-level model (Supplementary Note 5), we extract the lifetime of this SPE to be (2.07 ± 0.02) ns. The energy splitting between two BIC polaritons is clearly seen in the PL spectrum near normal incidence ($\theta \sim -$3.06°), which is fitted by a double-Lorentzian function (Fig. 3c). Furthermore, the polariton dispersions are modified from the original uncoupled cavity dispersion that is revealed by comparing the cross-sectioned PL spectra in the two cases (Supplementary Fig. 3b).

The extracted polariton energies and linewidths are plotted against the incident angle, as shown in Fig. 3d and e, respectively. The FWHM ($\gamma$) of the two polariton bands reduces and becomes identical when $\left|\theta \right|$~1.93°. The BIC polariton properties can be well modelled by using coupled oscillator theory (Supplementary Note 3). The calculated eigenvalues are shown by black lines, where the real parts are eigenenergies (Fig. 3d), and imaginary parts are FWHM of polariton branches (Fig. 3e). We used a detuning energy of 1 meV between the BIC resonance and the SPE at $\theta$ = 0. The Rabi splitting is then defined to be $\sim \,$ meV at $\left|\theta \right|$~1.24°, corresponding to a coupling strength $g$~2 meV. The vanishing of the signal at normal emission in both branches is the hallmark of polariton BIC that has been previously reported for the strong coupling regime between excitons in quantum wells and photonic BIC^28,39^. The spectral splitting, the narrowing of linewidth, the vanishing of the signal at normal incidence and the good agreement between experiment and theory confirm the strong coupling nature of the SPE and BIC in our systems.

Remarkably, the coupling strength $g$~2 meV in our system is one order of magnitude higher than the record established for the strong coupling regime using InAs/GaAs quantum dots at cryogenic temperatures^10–14^. Using Eq. (1), we deduce an oscillator strength per unit volume $f/V$ of two orders of magnitude higher than the one in InAs/GaAs systems. One possible way to acquire such a large $f$ is through the interaction of BIC modes with other midgap states via the polariton branches as theoretically proposed^40^. Nevertheless, further study, especially a density functional theory (DFT) calculation, is needed to unravel the underlying physics behind the large oscillator strength of this class of SPEs.

To further investigate the SPE-BIC coupling, we employed excitation power to detune the energy difference between the SPE and BIC mode. Specifically, when increasing the power density from 0.672 kW/cm^2^ to 25.784 kW/cm^2^, the BIC resonance energy increases by $\sim$2.2 meV, while the SPE peak remains unchanged (Supplementary Fig. 5). Therefore, the excitation power can be used to vary the detuning energy between SPE and BIC. Here, the detuning at $\theta=$ 0 is varied from 1.0 meV to 3.8 meV when increasing the excitation power. It is worth noting that the SPEs are optically stable under our experimental conditions.

**Fig. 4:**
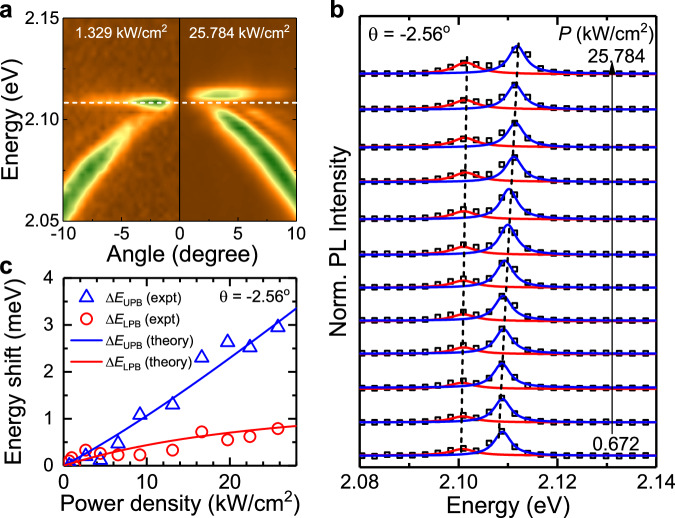
Tuning of strong coupling by excitation power. **a** Angle-resolved PL spectra acquired under different excitation power: ${P}_{{{{{{\rm{exc}}}}}}}$~1.3^2^9 kW/cm^2^ (left) and ${P}_{{{{{{\rm{exc}}}}}}}$~25.784 kW/cm^2^ (right) showing the blueshifts of both polariton branches with increasing power. The white dashed line is guideline. **b** Normalised PL spectra extracted at $\theta=$ −2.56° under varying excitation power density from 0.672 kW/cm^2^ (bottom) to 25.784 kW/cm^2^ (top), which induces changes in detuning energy and energy splitting. All spectra are fitted by double Lorentzian, and the constituents are shown by red and blue solid lines for LPB and UPB, respectively. **c** Energy shift of UPB ($\Delta {E}_{{{{{{\rm{UPB}}}}}}}$, blue triangles) and LPB ($\Delta {E}_{{{{{{\rm{LPB}}}}}}}$, red circles) relative to the lowest used power. Theoretical fitting curves are shown by solid lines considering the power-induced energy shift of the uncoupled SPE and BIC modes.

As a result of the varying detuning energy, we observe the energy shift of polariton branches, as shown in Fig. 4a for two representative excitation powers ${P}_{{{{{{\rm{exc}}}}}}}$~1.329 kW/cm^2^ and ${P}_{{{{{{\rm{exc}}}}}}}$~25.784 kW/cm^2^. The power-dependent PL spectra extracted at an angle $\theta \sim$ −2.56 collected at various excitation powers are shown in Fig. 4b, and the corresponding energy shifts of upper polariton branch (UPB) and lower polariton branch (LPB) are plotted as a function of excitation power in Fig. 4c. With increasing the excitation power, both polariton branches show blueshifts. Over the investigated power range, the two branches do not cross each other. At the highest power used, the UPB and LPB peak shifts by $\Delta {E}_{{{{{{\rm{UPB}}}}}}} \sim$ 3.0 meV and $\Delta {E}_{{{{{{\rm{LPB}}}}}}} \sim$0.8 meV, respectively. The observed energy shifts can be well reproduced by the coupled oscillator model (solid curves) by considering the power-induced changes in the detuning energy. The power-dependence of ${E}_{{{{{{\rm{UPB}}}}}}}$ and ${E}_{{{{{{\rm{LPB}}}}}}}$ further supports our attribution of the band splitting to the strong coupling. Indeed, the hybrid nature of mixed BIC-SPE in the two bands imposes the blueshift for both bands, with the shifting dictated by the BIC fraction.

## Discussion

We have experimentally realised, at room temperature, strong coupling between a single-photon emitter and a bound-state-in-the-continuum cavity. The absence of emission at normal incidence, along with the narrowing of bandwidths at BIC condition and the characteristic Rabi splitting between the BIC polariton branches of up to $\sim \,$ meV, have been resolved. Our work offers great opportunities to advance the fundamental understanding of quantum dynamical systems in strong coupling regime. Since SPEs in hBN are relatively new sources of quantum emitters, the fundamental understanding on their microscopic origins is still at early stage. Intriguingly, some types of SPEs in hBN exhibit room-temperature spin polarization^30^ that, when strongly couple to cavity photons, can be used as spin qubits. The development of such spin-photon interface platform could herald new paradigms in quantum information processing, notably with the integration of on-chip quantum photonic circuits. From our single cQED system, future work along the line of deterministically positioning SPEs in hBN and controlling their emission properties, such as wavelength, linewidth, polarisation and spin, will be the next critically important step to establish the entanglement between two or more cQED units that, subsequently, can be integrated into a more complex quantum system. Some viable routes could be using AFM indentation^41^, electron-beam irradiation^42^, direct growth of hBN on nanostructured metasurface^43^, two-dimensional van der Waals heterostructures^44^ and monolithic hBN metasurface structures. Furthermore, in comparison with other solid-state quantum emitters, our approach of using SPEs in 2D platforms enables the fabrication of advanced 2D quantum devices and hold potential to scale up towards practical applications at room temperature.

## Methods

### Nanofabrication

A 150-nm thick film of TiO_2_ was deposited onto a quartz substrate by ion-assisted deposition (IAD, Oxford Optofab3000), and then a 30-nm-thick film of Cr was deposited by electron-beam evaporation (Angstrom EvoVac) as a hard mask layer. A negative electron-beam resist, hydrogen silsesquioxane (HSQ, Dow Corning), was spin-coated on TiO_2_/Cr/quartz at 5000 rpm for 60 s, followed by baking at 120 °C for 2 min and at 180 °C for 2 min. The patterning was done with electron-beam lithography (Elionix ELS 7000), and the sample was developed using a salty developer (i.e., solution of 1 wt. % NaOH and 4 wt.% NaCl in DI water) for 4 min and rinsed with DI water^45^. The HSQ pattern was first transferred to Cr layer by using inductively coupled plasma reactive ion etching (ICP-RIE, Oxford Plasmalab 100) with a mixture of Cl_2_ gas at 15 standard cubic centimetres per minute (sccm) and O_2_ (2 sccm) gas at 10 mTorr and 7 °C. After that, Cr pattern was transferred into TiO_2_ film by using CHF_3_ gas using the same etching machine at 25 sccm, 32 mTorr and 5 °C. Finally, the whole sample was immersed in a chromium etchant solution (Merck) for 15 min to remove the remaining Cr and resist layers. The final sample was then rinsed in DI water and blow-dried with nitrogen gas.

### hBN film growth process

A few-layer hBN film was grown on a sapphire substrate by metal-organic vapour-phase epitaxy (MOVPE). More specifically, triethyl boron (TEB) and ammonia served as boron and nitrogen precursors, respectively. The sapphire substrate was initially functionalised with ammonia prior to hBN growth at a temperature of about 1000 °C. The chamber temperature was subsequently elevated to 1350 °C, and the precursors were introduced into the chamber once the temperature was stabilised. The precursors were intentionally injected into the reactor with short pulses lasting 1 to 3 s to minimise parasitic reactions.

### hBN film transfer process

Milimeter-sized hBN film was transferred from the growth sapphire substrate onto BIC structures using a wet transfer method. Approximately a 300-nm PMMA (A4, Mircochem) layer was spin-coated onto hBN/sapphire substrate and baked at 120 °C for 3 min to evaporate the polymer solvent. The sample was then floated onto a 1 M KOH aqueous solution to etch the sapphire interfacial layer and detach it from the hBN/PMMA film. After picking the floating film with the targeted substrate, the film was washed three times with DI water to remove the remaining base. A similar process was done to transfer hBN film onto the BIC structures. To gently remove water residue without the formation of wrinkles and bubbles, the substrate was placed in a vacuum desiccator for 30 min, followed by heating on a hotplate for another 30 min. This step is critical since some wrinkles are thick enough to interfere with the BIC modes. Thereafter, PMMA film was removed in a warm acetone bath overnight and then the sample was cleaned by a UV ozone (ProCleaner™ Plus, Bioforce Nanosciences Inc.) for 10 min to remove the remaining polymer. Finally, the hBN/BIC sample was annealed on a hotplate at 500 °C for 2 h before measurements to enhance adhesion between the film and substrate.

### Photoluminescence and photon autocorrelation measurements

The optical measurements of hBN SPEs were carried out on a home-built confocal microscope. We used a 532 nm continuous-wave laser for the excitation. Laser scanning was manipulated by an X-Y scanning mirror (FSM300^TM^, Newport Corp.). A 100× objective (Nikon, 0.9 NA) and one green dichroic mirror were used for the collection. Reflected laser and PL signals were filtered with an extra 568-nm long-pass filter. An additional bandpass filter centred at 587 nm with 15 nm bandwidth was used to only collect the emission from SPEs near resonance with the BIC mode. For detection, a flip mirror was used to guide the signal into a spectrometer (Princeton Instruments Inc.) or a single-mode fibre connected with two avalanche photodiodes (APDs) (Excelitas Technologies) via a 50:50 fibre splitter. For the polarisation-resolved PL, a rotating polariser was used to analyse the emission of SPEs, and the signal was detected by an APD. For the photon antibunching characterisation, the timing and correlation between two APDs were done by a correlator module (PicoHarp300^TM^). We recorded the coincidence count histogram with a 64-ps bin width resolution.

### Angle-resolved optical characterisations

Angle-resolved spectroscopic measurements were performed using an inverted optical microscope (Nikon Ti-U) coupled to a spectrometer equipped with an electron-multiplying charged-coupled detector (EMCCD, Andor Newton 971). For reflectance measurements, light from a halogen lamp was focused onto the sample surface via a 50× objective (Nikon, NA = 0.55) with a spot size of ~7 µm. For photoluminescence, the excitation was a 488 nm continuous-wave laser. The signals were collected by the same objective and passed through a series of lenses for back focal plane imaging. The wavelengths were resolved by a spectrograph (Andor SR-303i) with a single grating groove density of 150 gr/mm and a slit size of 100 µm.

### Supplementary information

Supplementary Information

Peer Review File

## Data Availability

The Source Data underlying the figures of this study are available at 10.6084/m9.figshare.25288324. All raw data generated during the current study are available from the corresponding authors upon request.

## Code availability

The codes for numerical simulation and data analysis presented in this manuscript are available from H.S.N. upon request.
