# Supplementary material for: Room-temperature strong coupling in a single-photon emitter-metasurface system
Source: Nat Commun. 2024 Mar 13;15:2281. doi: 10.1038/s41467-024-46544-w (PMC10937668; doi:10.1038/s41467-024-46544-w)
Supplement: Supplementary file 1 — Supplementary Information [file 41467_2024_46544_MOESM1_ESM.docx]

**Supplementary Information for**

**Room-temperature strong coupling in a single photon emitter-metasurface system**

T. Thu Ha Do^1,†^, Milad Nonahal^2,3, ǁ,†^, Chi Li^2,3,ǂ^, Vytautas Valuckas^1^, Hark Hoe Tan^4,5^, Arseniy I. Kuznetsov^1^, Hai Son Nguyen^6,7*^, Igor Aharonovich^2,3*^, Son Tung Ha^1*^

*Email: [hai-son.nguyen@ec-lyon.fr](mailto:hai-son.nguyen@ec-lyon.fr), [igor.aharonovich@uts.edu.au](mailto:igor.aharonovich@uts.edu.au), [ha_son_tung@imre.a-star.edu.sg](mailto:ha_son_tung@imre.a-star.edu.sg)

I. Supplementary Figures


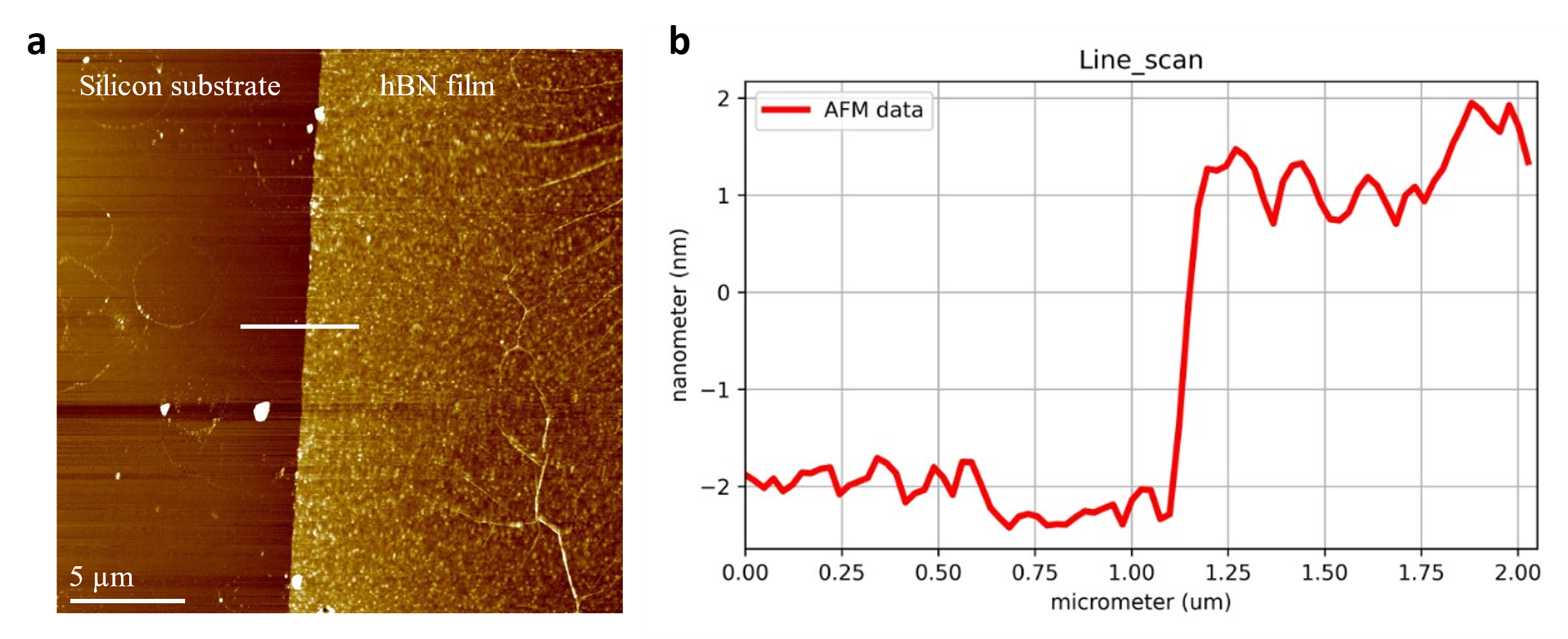


Supplementary Fig. 1| Atomic force microscopy (AFM) characterisation of hBN film. (a) AFM image of the as-grown hBN film on a sapphire substrate before transferring onto TiO_2_ nanostructures. (b) Height profile extracted along the white line in (a) revealing the film thickness of $\boldsymbol{\sim}$3 nm.


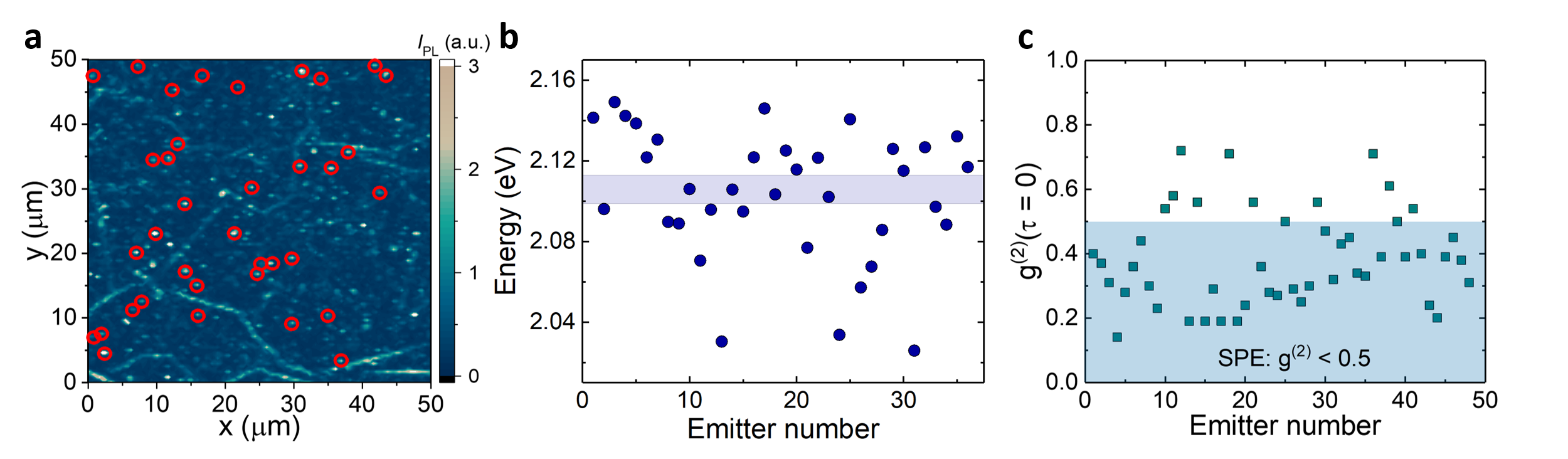


Supplementary Fig. 2| Characterisation of SPEs in hBN film. (a) Confocal PL map taken in a region of 50×50 μm^2^ on an hBN film, where 36 SPEs (with $g^{(2)}(0)$ < 0.5) were identified (red circles). (b) Statistics of emission energies showing only 4 (out of 36) SPEs emitting at $\boldsymbol{\sim}$2.106 eV (blue shaded-area), which match with the BIC resonance presented in Fig. 3 for strong coupling. (c) Statistics of $g^{(2)}(0)$ for all emitters detected by PL confocal setup. Single-photon emitters are defined as $g^{(2)}(0)$ < 0.5 (shaded area).


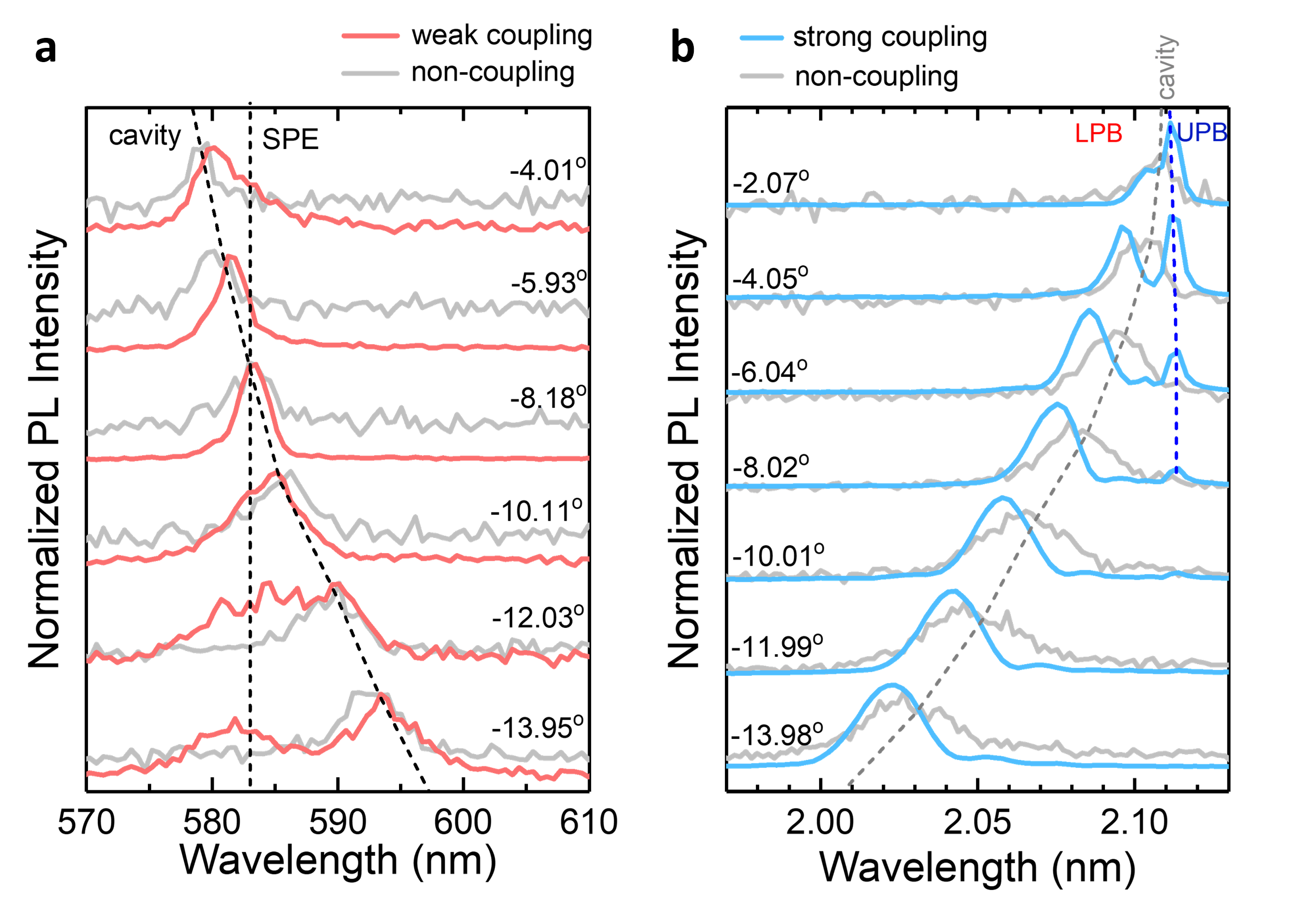


Supplementary Fig. 3| Band dispersions in weak and strong coupling cases. Comparison of PL spectra extracted at different angles for (a) weak coupling (solid red lines) versus non-coupling (solid grey lines) cases and (b) strong coupling (sky-blue solid lines) versus non-coupling (solid grey lines). In the weak coupling regime, the cavity mode dispersion remains unchanged, which is revealed by the overlap of PL peak of cavity modes with and without the presence of the SPE. The crossing between SPE and cavity bands is clearly resolved, which is the typical behaviour of weak coupling. In contrast, in a strong coupling regime, the cavity mode (grey dash-line) lies in between UPB (dark-blue dash-line) and LPB (red dash-line), indicating the change of band dispersion from the original cavity mode. Furthermore, the avoided crossing is observed when approaching normal incidence. These are the typical results from emitter-cavity strong coupling.


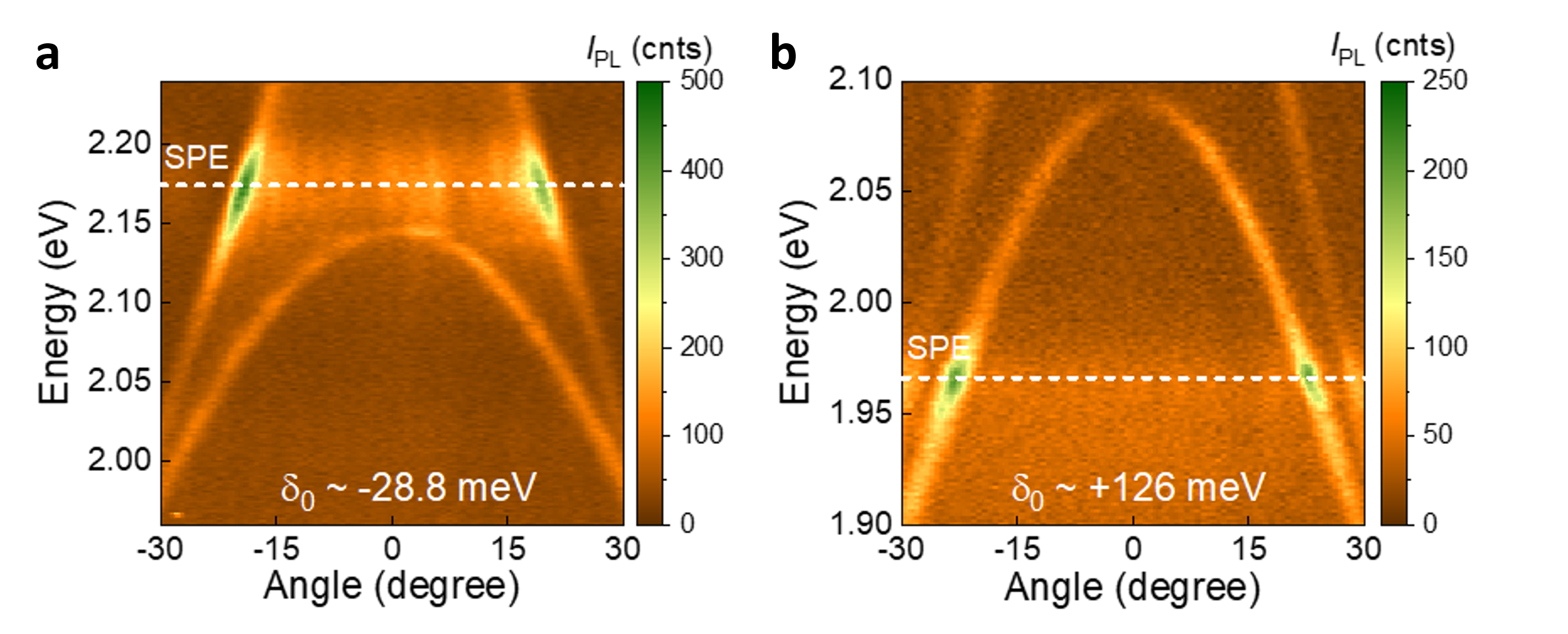


Supplementary Fig. 4| Weak coupling with large detuning energy. Angle-resolved PL spectra measured for different SPEs on different arrays for (a) negative detuning $\delta_{0}$ ~ -28.8 meV and (b) positive detuning $\delta_{0}$ ~ +126 meV. Due to the large energy mismatch, the SPEs only show weak coupling to resonance modes.


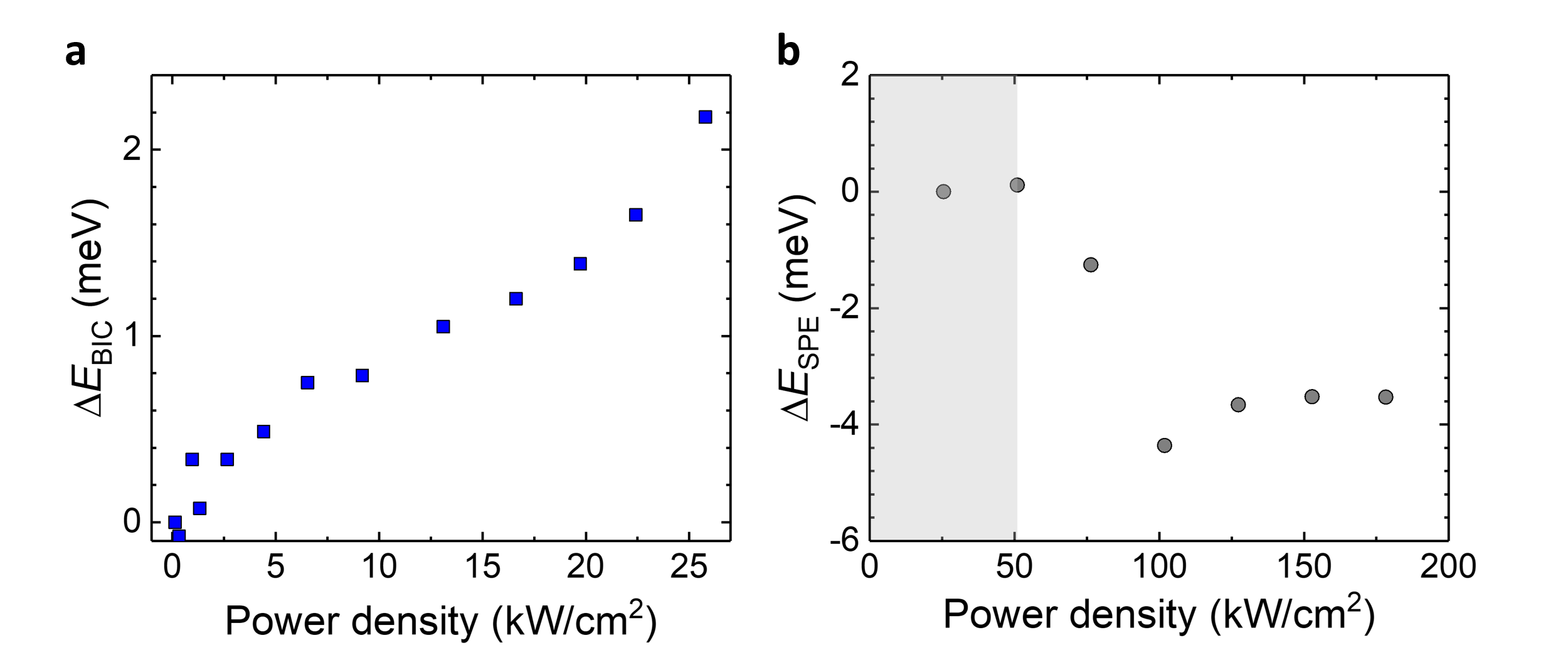


**Supplementary Fig. 5| Power-induced energy shift of BIC and SPE.** Energy shifts of (**a**) BIC ($\Delta E_{\mathrm{BIC}}$) and (**b**) SPE ($\Delta E_{\mathrm{SPE}}$) with varying excitation powers. When increasing power density from 0.672 kW/cm^2^ to 25.784 kW/cm^2^, the BIC resonance energy increases by ~2.2 meV, while the SPE peak remains unchanged (grey shaded region).

II. Supplementary Notes

**1. Dipole orientation**

We performed polarization-resolved experiments to measure the dipole orientation of SPEs. The photoluminescence (PL) signal from the SPEs was collected by an 100× objective (numerical aperture, NA = 0.9), analysed by rotating a polarizer ($\phi)$. The polarised emission was detected by an avalanche photodiode (APD). We further studied the polarisation of SPEs in two thin hBN films (sample 1 with thickness ~3 nm and sample 2 with thickness ~10 nm) and a thick film (thickness ~200 nm), as shown in Supplementary Fig. 6. The three-dimensional (3D) orientations of the dipoles can be derived from the degree of polarization (DOP) measured using the method described by Lethiec *et. al.*^1^.

For a linear dipole, which is our case, the degree of polarization is expressed as:

$$DOP=\frac{C\sin^{2} \beta}{\left( 2A-2B+C \right)\sin^{2} \beta+2B}, (1)$$

where $A, B, C$ are coefficients related to the sample configuration and objective numerical aperture (NA), and $\beta$ is the angle between the dipole orientation and *z*-axis. If the dipole is completely out-of-plane, *i.e.* $\beta=0$, DOP is equal 0, and the PL is unpolarised. When $\beta$ increases, DOP also increases, and the PL becomes more polarised in *x-y* plane. DOP reaches the maximum value of $C/(2A+C)$ when $\beta=90^{\circ}$ that is the case when the dipole is completely in-plane. For a large NA objective, the DOP is always less than unity regardless of $\beta$.

We found that all the investigated emitters exhibit a non-zero degree of linear polarisation with $DOP_{1} \sim$80% (for ~3-nm thick film), $DOP_{2} \sim$60% (for ~10-nm thick film) and $DOP_{3} \sim$30% (for ~200-nm thick film). The maximum polarisation induced by our optics is ~5%, which contributed to the experimental error of our results. Our results reveal that as the hBN film is thinner, the DOP increases that indicates the in-plane component of the dipole orientation $\mu_{//}$ becomes stronger. More importantly, for the case of sample 1 similarly to the sample used for strong coupling in the main text, the DOP of 80% is very close to the maximum DOP of 0.82 simulated for SPEs in hBN^2^, hence, the dipole in the ~3-nm thick film is approximately aligned in-plane.

Our results are in good agreement with other studies in the literature that the carbon-related defect SPEs in a few-layer-thin hBN film mostly aligned in plane^2,3^. This method is only valid in the case of a single dipole. For ensembles of emitters with random orientation, this method is not applicable.


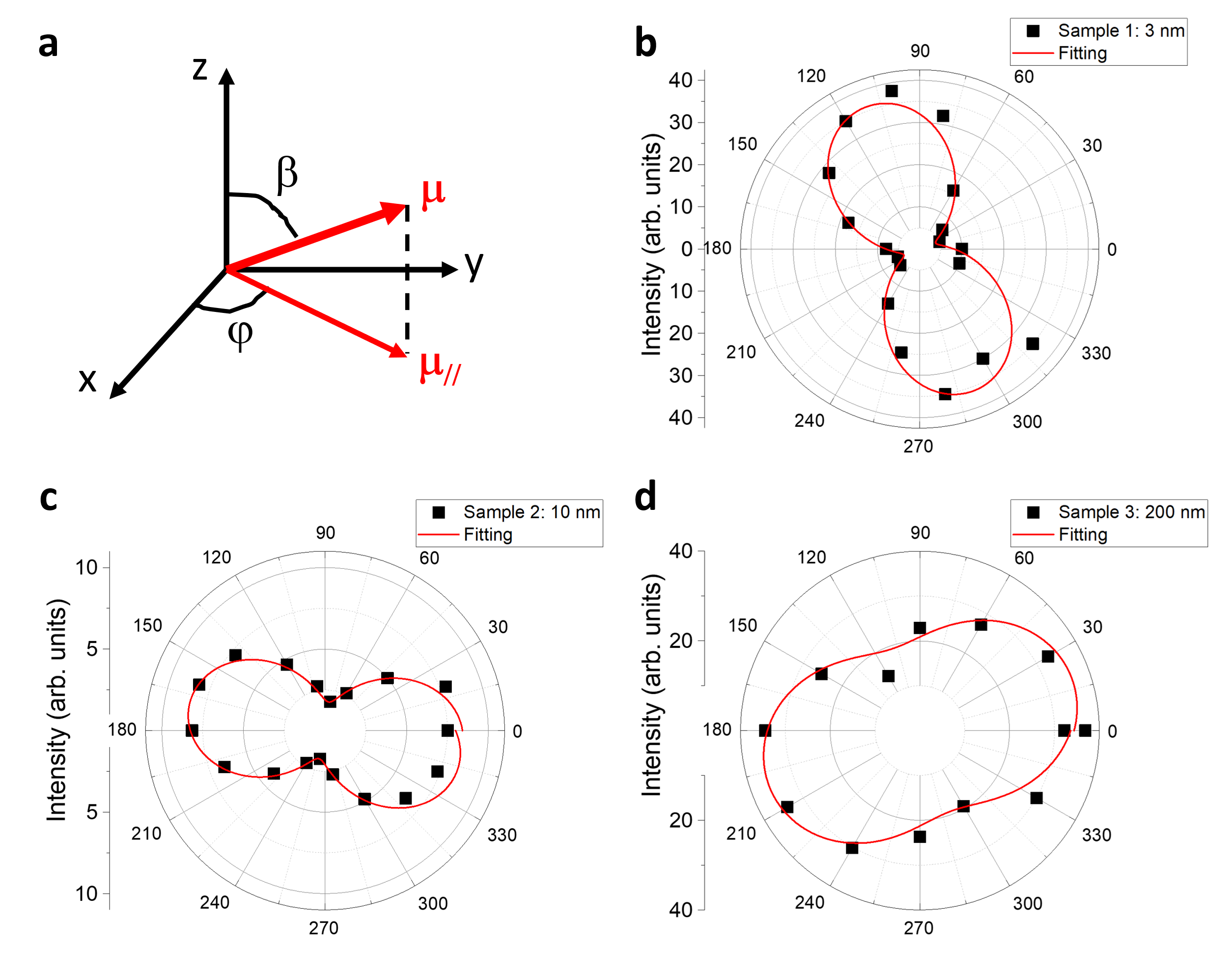


**Supplementary Fig. 6| Polarisation-resolved measurements to determine the dipole orientation.** (a) Schematic of an optical dipole $\mu$ in 3D space. The intensity was recorded by an APD while rotating the polariser in the collection path. Total intensity from SPEs versus $\phi$ for (b) sample 1 with thickness ~3 nm, (c) sample 2 with thickness ~10 nm and (d) sample 3 with thickness ~200 nm. All samples were grown by MOVPE method.

**2. Fraction of nanopillars contributing to strong coupling**

As discussed in the main text, the coupling strength $g$ is proportional to $\mu.E$, where $\mu$ is the transition dipole moment and $E$ is the electric field. The coupling strength is non-zero as long as $\mu$ and E are not perpendicular. A maximum coupling strength $g_{\max}$ is obtained when the dipole is located at the field maxima $E_{\max}$ and the dipole is perfectly aligned with the local field direction.

Given that the smallest linewidth reported for this kind of SPEs in hBN is 0.2 meV^[4]^ and the *Q*-factor of our BIC cavity is $1.3\times{10}^{5}$, the ultimate condition for strong coupling is $g\geq g_{\min}=(\kappa_{cav}-\kappa_{SPE})/2\approx0.1$ meV. The coupling strength resolved in our experiment is ~2 meV, which is 20 times larger than $g_{\min}$.

We estimate the coupling strength for a dipole located at a random location away from the maximum field spot $E_{\max}$ with a random orientation with respect to the local electric field as such:

$$\frac{g}{g_{\max}}=\frac{\left| \mu\right||E|\sin\beta\cos\alpha}{\left| \mu\right||E_{\max}|\sin\left( \frac{\pi}{2} \right)\cos0}=\frac{E}{E_{\max}}\sin\beta\cos\alpha, (2)$$

where $\beta$ is the angle between $\mu$ and *z*-axis ($\beta\in[0, \pi/2]$), and $\alpha$ is the angle between $\mu_{//}$ and $E$ ($\alpha\in[-\pi/2, \pi/2]$). The diagram is given in Supplementary Fig. 7 below.

From the polarisation analysis, the dipole orientation of the SPEs generated in a thin hBN film (~3 nm) is approximately in-plane (*i.e.*, large $\mu_{//}$ or large $\beta$) that is favourable for the coupling with the in-plane electric field. For simplicity, we will assume that the dipole is in-plane and substitute $\beta=\pi/2$ into equation (1) in the main text:

$$\frac{g}{g_{\max}}=\frac{E}{E_{\max}}\cos\alpha. (3)$$

From our calculation for electric field distribution, the area for $E/E_{\max}>0.5$ is ~68% of the array area (Supplementary Fig. 7c). In addition, the probability for $\cos\alpha>0.5$ (*i.e.*, $\alpha\in[-\pi/3, \pi/3]$) is 33% as $\alpha$ spans from $-\pi/2$ to $\pi/2$. Therefore, we estimate the probability for $g/g_{\max}>0.25$ is $0.68\times0.33=0.2244$ or $\sim22\%$.


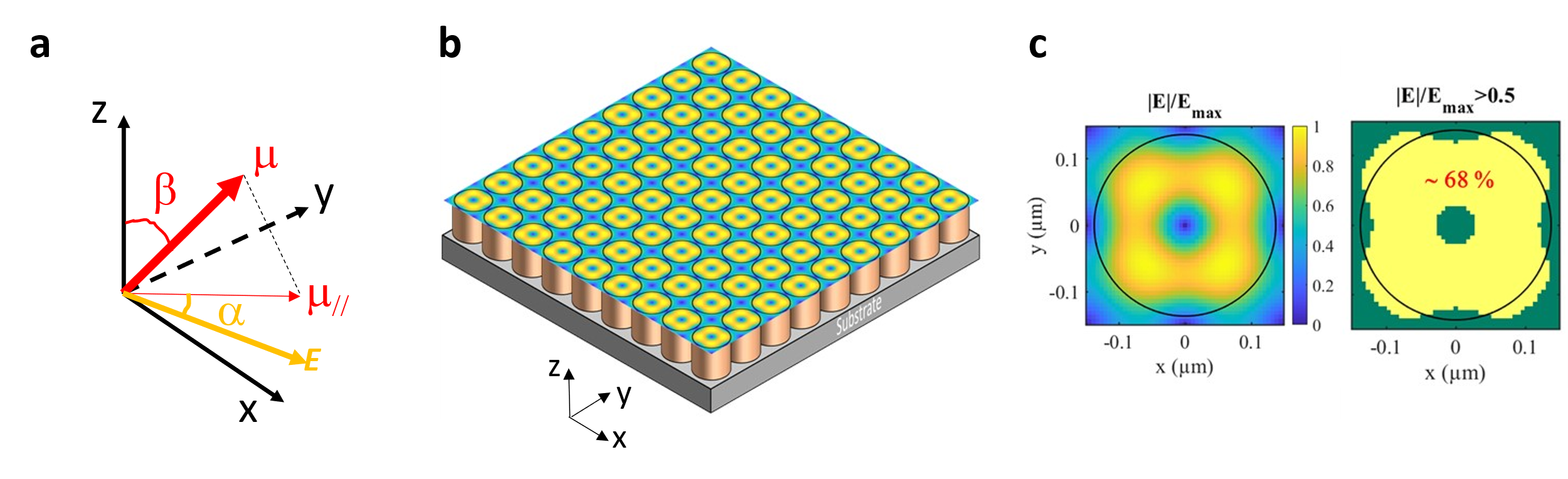


**Supplementary Fig. 7| Fraction of nanopillars contributing to strong coupling.** (a) Schematic of a single dipole orientation in 3D space. (b) Electric field distribution in the hBN film on top of TiO_2_ metasurface. (b) Electric field distribution on one representative pillar from top-view (*x-y* plane). (c) Highlighted area (yellow) where the magnitude of the electric field is 50% of its maximum value.

In summary, we estimate a probability of 22% that the coupling strength drops to 25% of its maximum possible value considering 50% drop in the electric field strength and 50% drop due to the dipole orientation. As compared to other types of 3D SPEs such as molecules or semiconductor quantum dots, our 2D-like SPEs in a 2D material like hBN reduce one more degree of polarization randomness ($\beta$) when incorporating in a resonant cavity.

**3. Coupled oscillator theory**

3.1. BIC models

The energy and losses of the BIC mode as functions of angle are modelled by:

$$E_{\mathrm{BIC}}\left( \theta\right)=E_{\mathrm{BIC}}\left( \theta=0 \right)+U-\sqrt{U^{2}+v^{2}{.k\left( \theta\right)}^{2}} (5)$$

and

$$\kappa_{\mathrm{BIC}}\left( \theta\right)=\left( \frac{1}{\kappa_{\infty}}+\frac{1}{\alpha{.k(\theta)}^{2}} \right)^{-1} (6)$$

where $k\left( \theta\right)=(2\pi/\lambda)\sin\theta$ is the in-plane wavevector, $v$ represents the group velocity of the BIC mode at high oblique angles, $\kappa_{\infty}$ is the losses of the BIC mode at high oblique angles. Importantly, the dependence $\alpha{.k(\theta)}^{2}$ represents the transformation from BIC at $\theta$ = 0 to quasi-BIC when a slightly oblique angle is introduced. Here, the experimental results are fitted using $U=0.3$ eV, $v=0.1$ eV.μm, $\kappa_{\infty}=20$ meV and $\alpha=30$eV.μm^2^.

3.2. Coupled oscillator models

The light-matter interaction is expressed as:

$H=\left( \begin{matrix} E_{\mathrm{BIC}}(\theta)+i\kappa_{\mathrm{BIC}}(\theta)/2 & g \\ g & E_{\mathrm{SPE}}(\theta)+i\kappa_{\mathrm{SPE}}/2 \end{matrix} \right), (7)$

where $E_{\mathrm{BIC}}(\theta)$ and $E_{\mathrm{SPE}}(\theta)$ are energy dispersion of the uncoupled BIC cavity and the SPE; $\kappa_{\mathrm{BIC}}(\theta)$ and $\kappa_{\mathrm{SPE}}$ represent loss of BIC and SPE modes (FWHM in emission spectra); $g$ is the coupling strength.

The complex eigenenergies of the Hamiltonian are:

$$\tilde{E}_{\pm}\left( \theta\right)=\frac{E_{\mathrm{BIC}}\left( \theta\right)+E_{\mathrm{SPE}}\left( \theta\right)}{2}+i\frac{\kappa_{\mathrm{BIC}}\left( \theta\right)+\kappa_{\mathrm{SPE}}}{4}\pm\sqrt{{\Delta(\theta)}^{2}+g^{2}} (8)$$

with $\Delta\left( \theta\right)=\frac{E_{\mathrm{BIC}}\left( \theta\right)-E_{\mathrm{SPE}}\left( \theta\right)}{2}+i\frac{\kappa_{\mathrm{BIC}}\left( \theta\right)-\kappa_{\mathrm{SPE}}}{4}$ .

The energies and linewidths of the polariton states are given by the real and imaginary components of the eigenenergies:

$$E_{\mathrm{UPB}/\mathrm{LPB}}\left( \theta\right)=\mathrm{Re}\left[ \tilde{E}_{\pm}\left( \theta\right) \right] (9)$$

$$\gamma_{\mathrm{UPB}/\mathrm{LPB}}=2\mathrm{Im}\left[ \tilde{E}_{\pm}\left( \theta\right) \right] (10)$$

The polariton states are eigenvectors of the Hamiltonian, given by:

$$|\left. \mathrm{UPB}/\mathrm{LPB} \right\rangle=\left| \left. \mathrm{BIC} \right\rangle+A_{\pm}\left( \theta\right) \right|\left. \mathrm{SPE} \right\rangle(11)$$

with $A_{\pm}\left( \theta\right)=\pm\sqrt{1+\left[ \frac{\Delta\left( \theta\right)}{g} \right]^{2}}-\frac{\Delta\left( \theta\right)}{g}$

The photonic (BIC) and excitonic (SPE) fractions of the polariton states are given by:

$$W_{\mathrm{UPB}/\mathrm{LPB}}^{BIC}\left( \theta\right)=\left( 1+\left| A_{\pm}(\theta) \right|^{2} \right)^{-1} (12)$$

$$W_{\mathrm{UPB}/\mathrm{LPB}}^{SPE}\left( \theta\right)=1-W_{\mathrm{UPB}/\mathrm{LPB}}^{BIC}\left( \theta\right) (13)$$

A blueshift $\delta_{BIC}$ of the BIC mode will lead to blueshifts of the polariton states that are given by:

$$\delta_{\mathrm{UPB}/\mathrm{LPB}}\left( \theta\right)=\frac{\delta_{BIC}}{2}\left\{ 1\mp\left( \left[ \frac{g}{\Delta\left( \theta\right)} \right]^{2}+1 \right)^{-1/2} \right\} (14)$$

One may show that the blueshift increases with superior BIC fraction in the polaritonic state.

**4. Estimation of quality factor**

As going closer to the normal incidence, the linewidth becomes narrower (Fig. 2d in the main text), and the optical intensity becomes weaker. At the BIC condition of the symmetry-protected BIC (Γ-point or $k=0$, where $k$ is the wavevector), the optical signal is completely absent (Fig. 2a in the main text). As a result, the resonance characteristics (energy, linewidth, intensity) cannot be reliably extracted from the experimental data. Therefore, the Q-factor (defined as $Q=Energy/FWHM$) exactly at the BIC condition (0$^{\circ}$) cannot be obtained from experiment. Within the resolution limit of our experimental setup, the smallest angle that we can resolve the optical mode is at ~$\pm$1.5$^{\circ}$. As moving away from normal incidence, the *Q*-factor drops significantly ($Q\sim1/k^{2}$). Therefore, the *Q*-factor value obtained at ~ $\pm$1.5$^{\circ}$ is still much lower than the value at 0$^{\circ}$.

In practice, due to the finite size ($50\times50$ μm^2^) of our metasurface, corresponding to $167\times167$ nanopillars, the BIC mode reduces to quasi-BIC with a finite *Q*-factor at normal incidence. To estimate the value of $Q_{BIC}$, we first calculate the *Q*-factor of the BIC band for the infinite structure. The simulation has been performed via the Rigorous Coupled Wave Analysis (RCWA) method. It shows that the *Q*-factor of the passive structure diverges when going to $k=0$, given by $Q\sim1/k^{2}$, as expected for the symmetry-protected BIC. Here, the $k^{-2}$ represents the decrease of the quality factor in the presence of a symmetry breaking at an oblique wavevector.


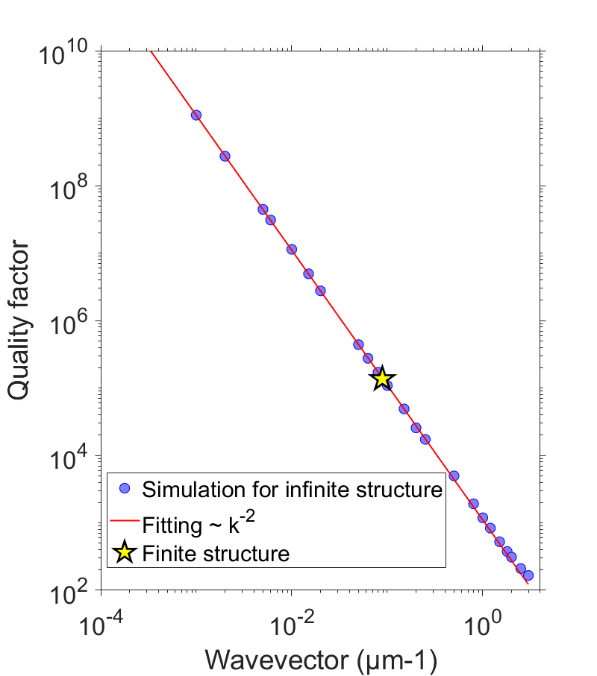


**Supplementary Fig. 8|** **Quality factor of BIC near normal incidence.** Theoretical calculation of quality factor for infinitely large metasurface (purple circles) which follow $Q\sim1/k^{2}$ by the fitting (red line). For a finite structure of $50\times50$ μm^2^, the Q-factor is determined to be $1.3\times{10}^{5}$ (yellow star).

For a finite size metasurface, the momentum space is now quantized as $\mathbf{k}_{n_{x},n_{y}}=n_{x}\pi/L\hat{\mathbf{x}}+n_{y}\pi/L\hat{\mathbf{y}}$ μm^-1^, where $n_{x}$, $n_{y}$ are positive integers and $L$ is the array length^5^. Consequently, the BIC becomes a quasi-BIC, with *Q*-factor given by $Q_{BIC}=Q(k_{1,1})$. Therefore, we estimate the $Q_{BIC} \sim1.3\times{10}^{5}$ for our finite array $50\times50$ μm^2^ (Supplementary Fig. 8).

**5. Lifetime fitting procedure**

From the $g^{(2)}$ presented in Fig. 3b, we extracted a lifetime value of (2.07 ± 0.02) ns for the emitter in the BIC cavity in strong coupling conditions. The extraction is done by a common $g^{(2)}$ fitting procedure as described in Ref.^6^. In specific, the $g^{(2)}$ data were first normalized to the flat region then fitted by a three-level model:

$$g^{(2)}=a-b\exp\left( -\frac{t}{\tau_{1}} \right) +c\exp\left( -\frac{t}{\tau_{2}} \right), (4)$$

where $\tau_{1}$ and $\tau_{2}$ are the two decay rates of the excited and metastable states, respectively; $a, b$ and $c$ are fitting parameters. Note that $\tau_{1}$ extracted from this $g^{(2)}$ fitting approaches the radiative lifetime when the pumping power $P$ is much lower than the saturation power $P_{sat}$^[6,7]^, which is typically higher than 500 μW and can be as high as 1500 μW for our SPEs (Supplementary Fig. 9). In our experiments, the used power is ~ 0.2$P_{sat}$.

**Supplementary Fig. 9| Saturation power of SPEs.** Power dependence of PL intensity of different carbon-related single-photon emitters in hBN (colored symbols) and the fitting with a saturation function $I=I_{0}P/(P_{sat}+P)$ showing the saturation power of these emitters are typically higher than 500 μW.

We characterised the radiative lifetime of other SPEs on the hBN film grown on SiO_2_/Si substrate of the same batch as the sample shown in the main text. Overall, $\tau_{1}$ ranges from 1.5 to 5.5 ns, while a majority of them have a lifetime of less than 3 ns (Supplementary Fig. 10) that are consistent with previous studies^8^. The shorter lifetime of the SPEs indicates the stronger brightness and a stronger oscillator strength that would be more likely to exhibit strong coupling.


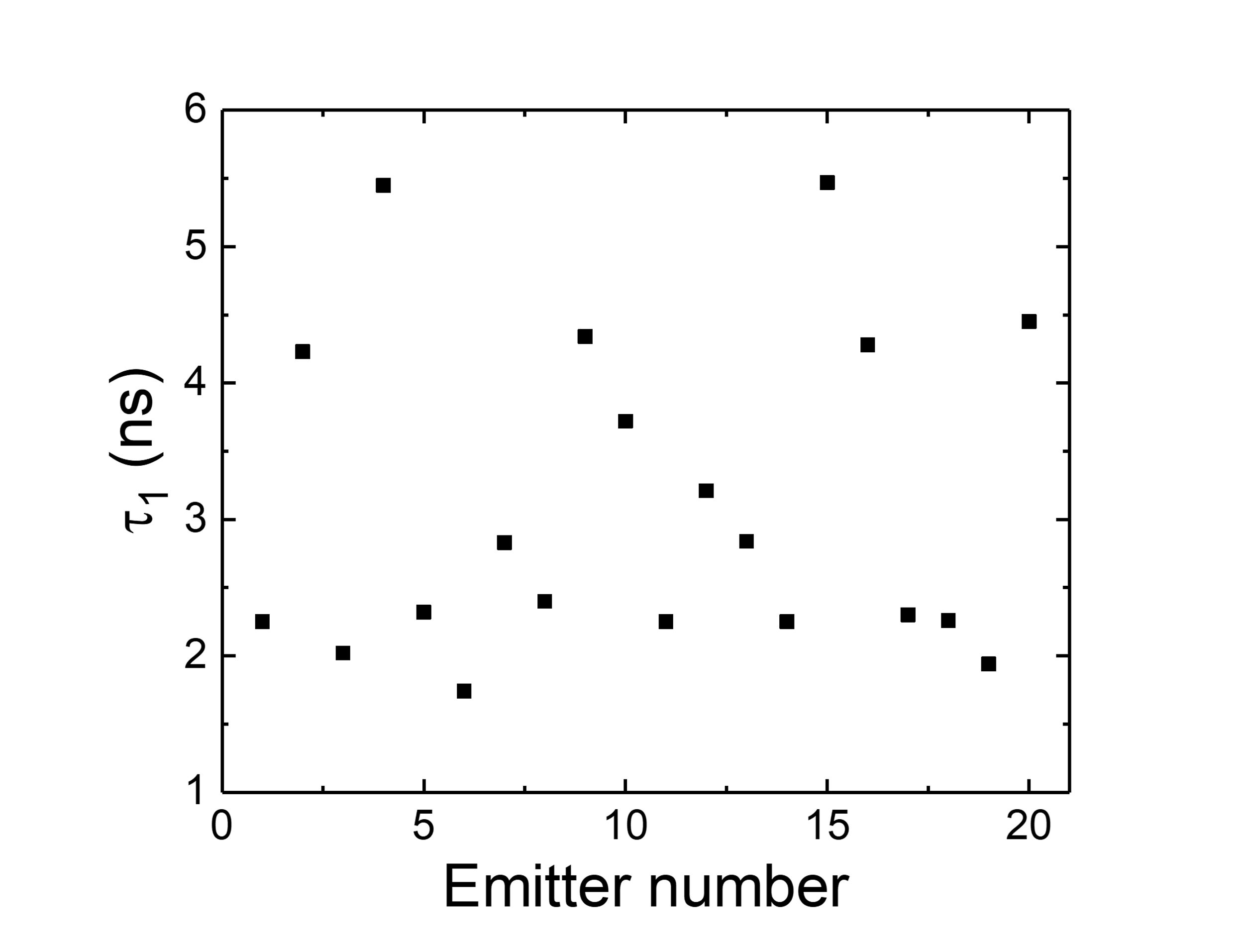


**Supplementary Fig. 10| Lifetime (**$\boldsymbol{\tau}_{\boldsymbol{1}}\boldsymbol{)}$ **of SPEs in hBN film on SiO_2_/Si substrate.** A majority of the emitters show lifetime of less than ~3 ns.

The fitting parameters to the $g^{(2)}$ at strong coupling condition (Fig. 3b in the main text) are $a=(0.95 \pm0.01)$, $b=(0.87 \pm0.03)$, $c=(0.20 \pm0.01)$, $\tau_{1}=(2.07 \pm0.02)$ ns and $\tau_{2}=(460.29\pm43.86)$ ns. We attribute the shorter decay component $\tau_{1}$ to the radiative lifetime of the emitters, and the long-decay component $\tau_{2}$ is likely related to non-radiative decay processes or decays of metastable states.

**Supplementary References**

1. Lethiec, C. *et al.* Measurement of Three-Dimensional Dipole Orientation of a Single Fluorescent Nanoemitter by Emission Polarization Analysis. *Phys. Rev. X* **4**, 021037 (2014).

2. Nikolay, N. *et al.* Direct measurement of quantum efficiency of single-photon emitters in hexagonal boron nitride. *Optica* **6**, 1084 (2019).

3. Takashima, H. *et al.* Determination of the Dipole Orientation of Single Defects in Hexagonal Boron Nitride. *ACS Photonics* **7**, 2056–2063 (2020).

4. Dietrich, A., Doherty, M. W., Aharonovich, I. & Kubanek, A. Solid-state single photon source with Fourier transform limited lines at room temperature. *Phys. Rev. B* **101**, 081401 (2020).

5. Zhou, M. *et al.* Increasing the *Q* -Contrast in Large Photonic Crystal Slab Resonators Using Bound-States-in-Continuum. *ACS Photonics* **10**, 1519–1528 (2023).

6. Kurtsiefer, C., Mayer, S., Zarda, P. & Weinfurter, H. Stable Solid-State Source of Single Photons. *Phys. Rev. Lett.* **85**, 290–293 (2000).

7. Berthel, M. *et al.* Photophysics of single nitrogen-vacancy centers in diamond nanocrystals. *Phys. Rev. B* **91**, 035308 (2015).

8. Jungwirth, N. R. & Fuchs, G. D. Optical Absorption and Emission Mechanisms of Single Defects in Hexagonal Boron Nitride. *Phys. Rev. Lett.* **119**, 057401 (2017).
